# COVID-19 and coronaviral hepatitis: evidence of collateral damage

**DOI:** 10.2217/fvl-2020-0065

**Published:** 2020-06-12

**Authors:** Mohammad K Parvez

**Affiliations:** ^1^Department of Pharmacognosy, King Saud University College of Pharmacy, Riyadh, Saudi Arabia

The newly identified severe acute respiratory syndrome coronavirus-2 (SARS-CoV-2) that originated in December 2019 in Wuhan, China, and caused a pandemic of coronavirus disease (COVID-19) has been declared a global public health emergency [1]. After SARS-CoV-1 and the Middle East respiratory syndrome-CoV (MERS-CoV), it is the third highly pathogenic betacoronavirus that, at the time of writing, has infected over 4,137,190 people and caused about 285,760 deaths worldwide [1]. Though, the first source of origin, high transmission and mechanisms of severity of SARS-CoV-2 in humans are hitherto not clearly established, its close resemblance (~96% identity) with bat SARS-like coronaviruses (bat-SL-CoV) RNA sequences has been recently confirmed [2]. The SARS-CoV-2 genome is a single-strand positive-sense RNA (∼30 kb) with 5′-cap and 3′-poly(A) tail, and consists of 12 open-reading frames that translate into various structural, nonstructural and accessory proteins, essential for its life cycle and pathogenesis [2,3]. Of these, the viral structural ‘spike/S’ protein’s C-terminal structural subunit ‘S1’ binds to the human cell-receptor Angiotensin Converting Enzyme-2 whereas the ‘S2’ subunit is required for cell membrane fusion [3].

SARS-CoV-2 has the incubation period of 2–14 days with symptoms of fever, cough, headache and breathlessness, manifesting from mild pneumonia to severe illness and death. In addition, some patients may also experience rashes on toes, discoloration of skin, dizziness, fizzing, burning sensation and loss of taste or smell. COVID-19 patients mostly in old age or with pre-existing chronic conditions like respiratory, cardiac, renal and hepatic disorders have shown higher mortality rate. The human-to-human transmission of COVID-19 has been confirmed through multiple modes, such as nasal droplets, aerosols and oral mucus [4].

## COVID-19 & digestive symptoms

Further studies have identified the potential transmission routes of SARS-CoV-2 through blood and feces samples of COVID-19 patients. The SARS-CoV-2 RNA has been detected in anal or rectal swabs and blood of hospitalized COVID-9 patients in China in a later stage of infection [5]. Of the 15 patients tested, eight were oral swabs positive (53.3%), four were anal swabs positive (26.7%), six blood positive (40%) and three serum positive (20%). Although, two patients were both oral and anal swab positive, none of the blood positive samples was swabs positive. Notably, anal swabs were found more test positive than oral swabs, suggesting the potentiality of fecal–oral transmission of SARS-CoV-2 [5]. Further, in a clinical investigation of ten pediatric COVID-19 confirmed cases, rectal swabs of eight children (80%) were persistently tested RNA positive even after nasopharyngeal test was negative, presenting the evidence of viral shedding through the GI tract [6]. In another study from China, stool specimens of COVID-19 patients were found SARS-CoV-2 positive, even after the clearance of the virus [7–9]. Nonetheless, detection of SARS-CoV-2 RNA cannot always be correlated with the presence of infectious virus particles in such samples. In clinical studies, a proportion of COVID-19 patients have shown evidence of gastrointestinal symptoms, and of these about 10% of hospitalized patients had diarrhea, nausea, vomiting and abdominal pain within 1–2 days before onset of fever and dyspnea [10].

## Coronaviral hepatic pathogenesis

SARS-CoV-2 has been linked to mild-to-moderate liver injury as revealed by elevated serum aminotransferases (ALT/AST), bilirubin, hypoproteinemia and prothrombin time prolongation, supported by liver histopathology [11–14]. Single-cell RNA sequencing data from two distinct cohorts of COVID-19 patients have shown elevated expression of Angiotensin Converting Enzyme-2 receptor in cholangiocytes (59.7%) than hepatocytes (2.6%), indicating that SARS-CoV-2 might directly affect intrahepatic bile ducts [15]. In a recent clinical study of 194 COVID-19 patients, 30 patients (15.46%) showed liver dysfunction [16]. Although in some COVID-19 cases, mild derangement of liver function was observed, patients with different durations of symptoms showed no evidence that were later associated with greater liver function derangement. Although in a COVID-19 case, the postmortem liver biopsy showed only microvesicular steatosis [14], COVID-19 patients presented with digestive issues before respiratory problems had a higher risk of mortality compared with those without digestive symptoms. Moreover, in severe cases of COVID-19, liver dysfunction is also observed with greater activation of coagulative and fibrinolytic pathways along with altered platelets, neutrophil and lymphocytes profiles [11]. Moreover, chronic liver disease patients with impaired immunity because of classical hepatitis viruses (HBV, HCV, HDV and HEV) or other hepatotropic viruses (HGV, GBV, TTV and SENV) infection or nonalcoholic fatty liver disease/nonalcoholic steatohepatitis are more susceptible to COVID-19, and may present worse outcomes from acute respiratory distress syndrome compared with the other critically ill patients.

Notably, other respiratory viruses induce elevations of liver function biomarkers, very likely related to liver inflammation or hepatocytes damage as a result of interacting cytotoxic T-cells and Kupffer cells [17]. In line with this, SARS-CoV-2 is also proposed to cause viral hepatitis while inducing a dysregulated innate immune response. SARS-CoV-2-encoded nonstructural and accessory proteins are suggested to modulate induction of cellular interferon and cytokines, enabling the virus to evade antiviral mechanism of interferon-stimulated genes [18]. In addition, the host-immune responses through inflammatory and cytotoxic lymphocytes activities are critical to inhibiting viral replication and dissemination. Therefore, the immune overdrive along with cytolytic effects, results in disease severity. In a recent study of COVID-19 patients during recovery stage, the high ratio of classical CD14^++^ cells with increased inflammatory gene expressions as well as a greater abundance of CD14^++^IL1β^+^ cells was found [19]. Also, while the levels of CD4^+^ and CD8^+^ T cells were decreased significantly, inflammatory genes were highly expressed in these patients. In addition, the plasma B cells were found to be remarkably increased as compared to decreased level of naive B cells. Notably, several novel B-cell-receptor isotypes (IGHV3–15, IGHV3-30 and IGKV3-11), which were previously used for other virus vaccine development were also identified in COVID-19 patients. Furthermore, IL-1β and macrophage-colony stimulating factor (M-CSF) were predicted as novel candidate target genes for inflammatory storm whereas TNFSF13, IL-18, IL-2 and IL-4 seemed to be beneficial for the recovery of COVID-19 patients [19].

## Current diagnostics & treatment options

The WHO has recommended case definitions for COVID-19 where a positive case is one with a laboratory confirmation of SARS-CoV-2 infection, irrespective of clinical manifestations [1]. Currently, serology or antibody-based rapid-test kits and reverse-transcription PCR are the diagnostic tools to identify SARS-CoV-2 infected people. Chest x-ray and CT scan are the clinical method of noninvasive pulmonary assessment. Nonetheless, reverse-transcription PCR testing of rectal swabs and stool samples of COVID-19 patients along with naso-/oropharyngeal swabs, sputum, tracheal aspirate or bronchoalveolar lavage are highly recommended [20].

As part of ‘solidarity’ clinical trials for COVID-19 treatment, mainly chloroquine and hydroxychloroquine, the antimalarial drugs; remdesivir, the anti-Ebola virus drug; and azithromycin, an antibiotic, are under clinical investigations in several countries. There are, however, safety concerns that both may cause cardiotoxicity with prolonged use in patients with pre-existing chronic conditions like renal failure and hepatic disease [21,22]. Very recently, remdesivir has been granted emergency use authorization by the US FDA, clearing the way for its broader use in COVID-19 patients [23].

Nonetheless, though they have a small sample size, case studies of COVID-19 patients with liver issues suggest focusing on immune modulators besides antiviral trials. The IL-6 has a therapeutic role in both the innate and adaptive immune responses that protect the host from a variety of infections [24]. Notably, considerable release of IL-6 has been previously reported in SARS-CoV-1 and MERS-CoV patients and suggested to play a role in diseases severity [25,26]. In COVID-19 patients, a large number of T lymphocytes and mononuclear macrophages are activated, producing IL-6. Thereafter, IL-6 binds to the IL-6 receptor on the target cells, which causes the cytokine-storm and severe inflammatory responses in lungs and other organs [27]. Tocilizumab, a recombinant humanized anti-human IL-6 receptor monoclonal antibody, efficiently binds to the IL-6 receptor and blocks IL-6 inflammatory pathway of cell damage. Very recently, tocilizumab treatment showed normalization of decreased peripheral lymphocytes counts in 52.6% (10/19) of 85% (17/20) critically ill COVID-19 patients without obvious adverse reactions [27]. Also, in 84.2% (16/19) of patients, it significantly decreased the abnormally elevated C-reactive protein, showing tocilizumab as a promising treatment in driving the overactive inflammatory response in the SARS-CoV-2 infected lungs and other tissues. The FDA has also approved a Phase III clinical trial for evaluating tocilizumab in hospitalized patients with severe COVID-19 pneumonia [28]. Sarilumab, which is another IL-6 receptor antagonist, has also launched Phase II/III clinical trial to evaluate its efficacy in patients with severe COVID-19 infection [28]. In addition, the IL-1 receptor antagonist, Anakinra and the JAK1/2 inhibitor, baricitinib are currently under evaluation for COVID-19 [29].

## Control & prevention

Developing a vaccine is the best preventive measure, but it is also the most complicated process that may require 24–30 months. Nonetheless, quick initiatives have been already taken, and the two COVID-19 vaccines are in Phase I clinical trial in USA, one in UK and three in China [30,31]. Large scale Phase II and Phase III trials are warranted to determine its optimal required dose, its efficacy in elderly people and minimal side-effects. Moreover, fairly high levels of protective antibodies called ‘herd-immunity’ are produced in COVID-19 patients. Though, this acquired immunity and its longevity are still poorly understood, its current use as plasma therapy seems promising. Further, coronaviruses tend to be cold-seasonal that wane when temperature rises. That may also be true for SARS-CoV-2, but seasonal variations might not sufficiently slow this when it has so many immunologically naive hosts. In the meantime, the first and most important measure is to immediately quarantine the COVID-19 positive or suspected individuals while enforcing public safety guidelines on social distancing, use of sanitizers, masks and gloves. In the present pandemic situation, lockdown of cities, suspending domestic and international travels and sealing of international borders are enforced in several countries.

## Concluding remarks

There is a growing understanding of COVID-19 pathobiology and SARS-CoV-2 virology, epidemiology and clinical management strategies. However, as evidenced by the recent clinical studies, even if the oral or nasal swabs are SARS-CoV-2 RNA negative, the anal or rectal swab, feces and blood samples can be positive and the patient might still remain viremic. The viral shedding in such samples there by, provides a cautionary warning that COVID-19 may be transmitted through oral-fecal route in developing countries with poor sanitization facilities, and most importantly, presence of SARS-CoV-2 in rectal, fecal and blood samples strongly endorse its gastrointestinal and hepatic etiology, directly or indirectly, in higher risk COVID-19 patients with impaired immunity. In the absence of an approved antiviral therapy, modulation of innate immune dysfunction should be, therefore, helpful in managing such group of patients. Nonetheless, by the time a vaccine is made available or the tempting ‘herd immunity’ scenario quickens, COVID-19 would terribly cost millions of lives and leave behind a trail of devastated health systems. Therefore, the world’s biomedical, socioeconomical and geopolitical forces must work together to bring the end of the COVID-19 emergency soon.
